# Emotion categorization of body expressions in narrative scenarios

**DOI:** 10.3389/fpsyg.2014.00623

**Published:** 2014-06-30

**Authors:** Ekaterina P. Volkova, Betty J. Mohler, Trevor J. Dodds, Joachim Tesch, Heinrich H. Bülthoff

**Affiliations:** ^1^Human Perception, Cognition and Action, Max Planck Institute for Biological CyberneticsTübingen, Germany; ^2^Graduate School of Neural and Behavioural SciencesTübingen, Germany; ^3^Department of Brain and Cognitive Engineering College of Information and Communication, Korea UniversitySeoul, Korea

**Keywords:** emotion perception, emotional body expression, biological motion, motion capture, animation

## Abstract

Humans can recognize emotions expressed through body motion with high accuracy even when the stimuli are impoverished. However, most of the research on body motion has relied on exaggerated displays of emotions. In this paper we present two experiments where we investigated whether emotional body expressions could be recognized when they were recorded during natural narration. Our actors were free to use their entire body, face, and voice to express emotions, but our resulting visual stimuli used only the upper body motion trajectories in the form of animated stick figures. Observers were asked to perform an emotion recognition task on short motion sequences using a large and balanced set of emotions (amusement, joy, pride, relief, surprise, anger, disgust, fear, sadness, shame, and neutral). Even with only upper body motion available, our results show recognition accuracy significantly above chance level and high consistency rates among observers. In our first experiment, that used more classic emotion induction setup, all emotions were well recognized. In the second study that employed narrations, four basic emotion categories (joy, anger, fear, and sadness), three non-basic emotion categories (amusement, pride, and shame) and the “neutral” category were recognized above chance. Interestingly, especially in the second experiment, observers showed a bias toward anger when recognizing the motion sequences for emotions. We discovered that similarities between motion sequences across the emotions along such properties as mean motion speed, number of peaks in the motion trajectory and mean motion span can explain a large percent of the variation in observers' responses. Overall, our results show that upper body motion is informative for emotion recognition in narrative scenarios.

## 1. Introduction and related research

Emotion is an integral part of human-human interaction. During communication, we receive and transmit emotional information through many channels: prosody, facial expressions, word choice, posture, and body motion. The human body is often perceived as a tool for actions (e.g., walking, grasping, and carrying), but it is also an important medium for emotional expression (De Meijer, 1989; de Gelder et al., 2010). During communication, body motion can highlight and intensify emotional information conveyed by other channels (e.g., hitting the table with a clenched fist while expressing anger with the voice and the face), add extra nuances of meaning to emotional expressions (e.g., bowing slightly while greeting someone to show respect), or contrast emotional information coming from other channels (e.g., crossing your arms while saying “This is just great.” implies that you are actually displeased).

The research on emotional body language is particularly challenging because of the complexity of biological motion, since the human body has hundreds of degrees of freedom and can be used for action and emotion expression simultaneously. Here we will briefly mention the research most relevant to our work, for a more detailed and comprehensive survey please see the survey by Kleinsmith and Bianchi-Berthouze (2013). Earlier studies of biological motion mostly relied on still frame or video recordings for stimulus generation. Johansson (1973) developed now widely used technique of biological motion representation that retains motion information but eliminates form information. The moving figure is marked by a small number of illuminated points or stripes, that are positioned at the main body parts and joints. In the resulting point-light stimuli only these bright marks are visible to the observer. Such stimuli are strongly degraded, and so the identity of initial actors, as well as their age, gender, and body shape are hidden from the observer. The following years have seen point-light technique frequently applied in research on perception of biological motion, including emotion recognition studies. Some of the earlier studies concentrated on emotion perception from dance (Walk and Homan, 1984; Dittrich et al., 1996; Brownlow et al., 1997). Many studies have investigated the recognition of human actions (Pollick et al., 2001a) and intentions (Manera et al., 2010), identity (Loula et al., 2005), gender (Troje, 2002; Pollick et al., 2005; Brooks et al., 2008) and emotion (Pollick et al., 2001b; Atkinson et al., 2004; Clarke et al., 2005; Beck et al., 2012; Ennis and Egges, 2012) from biological motion using point-light displays. These studies showed that this degraded representation of the body motion still conveys enough information for the observers to accurately recognize the stimuli.

Not only is biological motion itself a complex phenomenon, the factors that influence emotional perception and expression in the body motion are numerous and often interact with each other. For instance, gender of the observer has an effect on emotion recognition accuracy, as well as the gender of the performer of the motion. In several studies it has been shown that female participants are better at recognizing neutral or negatively colored actions (Sokolov et al., 2011), especially if the actor is male, while male participants recognize positive emotions in body language expressed by female actors with high accuracy (Krüger et al., 2013).

According to Giese and Poggio (2003) there are two distinct neural mechanisms in the brain that facilitate recognition of biological information: one for motion, another for form. Atkinson et al. (2007) determined that both form and motion signals are important for the perception of affect from the body motion. Using point-light biological motion stimuli Heberlein et al. (2004) have further investigated the neural systems involved in emotion recognition in normal population and subjects with brain damage. While biological motion per se and emotional body expressions are understandably not one and the same thing, correlation has been found between a subject's ability to discern emotional cues from point-light displays and the subject's ability to discriminate biological from non-biological motion. This observation that differences in emotion recognition may be related to more basic differences in processing biological motion per se are supported by studies for typically developed adults (Alaerts et al., 2011) and for participants with Aspergers Syndrome Condition related atypicalities (Nackaerts et al., 2012). A detailed review on the tight connection between the processing of biological motion and social cognition, and hence, disturbances in both these aspects of human mind due to atypical development (Aspergers Syndrome Condition, Down Syndrome, pre-term birth) can be found in Pavlova (2012).

Studies on emotional body language have also investigated various aspects of emotion expression through body motion, such as how emotions modulate various actions, like walking (Roether et al., 2010) or knocking (Pollick et al., 2001b). Other research used general non-verbal portrayals of emotions (Atkinson et al., 2004; McDonnell et al., 2009; Kleinsmith et al., 2011; Beck et al., 2012), but the actors were still well aware that their body motion was of primary interest to the researchers since the tracking technology was focused on the body by, e.g., using full body suits, covering the face by a mask, restricting finger movement (Atkinson et al., 2004; McDonnell et al., 2009). Even though the used setups are completely justified by the research questions pursued in the related studies, such restrictions are very likely to prevent actors from expressing emotions in a natural way that would be typical of normal human-human interactions.

Our research aim was to investigate human perception of emotional body expressions that were captured in narrative settings, naturalistic yet well-controlled. For this we gathered a large dataset of motion patterns of the upper body using a non-restrictive inertial body capture suit (Volkova et al., unpublished). The motion patterns served further as stimuli in emotion recognition experiments. We also argue that it is valuable to use a rich set of emotion categories for the categorization process. We conducted two perceptual experiments that evaluate the emotion recognition accuracy and consistency based exclusively on the upper body motions. Motivation behind focusing on upper body motion came partially from previous research by Glowinski et al. (2011), who successfully used videos of upper body emotional expression from the Geneva Multimodal Emotion Portrayal Corpus (Bänziger and Scherer, 2010) to cluster recordings across the valence-arousal dimensions. Additionally, focusing on upper-body motion allowed us to let the actors be seated during the motion capture sessions, a pose more common for narration situations in daily life, which in turn benefited our data recording and post-processing setups.

According to our null-hypothesis, the amount of information expressed through the body alone during narration should not be sufficient for an observer to recognize the emotion, since most of the information is expressed through the facial expressions, the speech prosody and, importantly, verbal content. However, our results show that even using stick figure stimuli and a large number of emotion categories, the recognition accuracy was above chance level, suggesting that upper body motion produced during narrative scenarios is informative for emotional categorization. Moreover, the responses from the observers are highly consistent and the agreement between participants was rather high according to Kendallas coefficient of concordance. Finally, we evaluated how much variance in the categorization performance could be explained by motion statistics of the stimuli.

## 2. Materials and methods

### 2.1. Motion sequences acquisition

Eight amateur actors were asked to perform a variety of natural emotionally expressive tasks. The motion sequences are distributed across the following eleven emotion categories: five positive (*amusement, joy, pride, relief, surprise*), five negative (*anger, disgust, fear, sadness, shame*), and *neutral*. Table 1 shows the number of motion sequences in descending order, representing intended emotion categories and acting tasks with the total number of motion sequences amounting to 1700. The motion was captured with the help of an Moven Xsens suit (Roetenberg et al., 2009) at the rate of 120 frames per second.

**Table 1:** **Count of motion sequences across emotion categories and acting scenarios**.

	**Short scenarios**				**Narrations**
	**Non-verbal**		**Sentences**		
**Emotion**	**Solitary**	**Communicative**	**Without direct speech**	**With direct speech**	
Joy	8	8	8	8	237
Sadness	8	8	8	8	200
Surprise	8	8	8	8	167
Pride	8	8	8	8	137
Anger	8	8	8	8	133
Neutral	0	0	0	0	116
Fear	8	8	8	8	112
Disgust	8	8	8	8	106
Relief	8	8	8	8	80
Amusement	8	8	8	8	59
Shame	8	8	8	8	33
Subtotal	80	80	80	80	
Total	320				1380
Grand total	1700				

*Two major types of acting tasks were short scenarios and narrations. The former were of two kinds: nonverbal (solitary or social) and short sentences (without direct speech or with)*.

Each actor came separately for four motion capture sessions, thus amounting to 32 motion capture sessions in total. In the first session each actor received four blocks of short scenarios to act out: *solitary non-verbal scenarios*, where the actor was instructed that they were to imagine they were alone; *communicative non-verbal scenarios*, where the actor was instructed to imagine they were in company of one or more people they knew; *short sentences without direct speech*, meaning only narrator's text was present; and finally *short sentences with direct speech*, where narrator's as well as a story character's text were present. In each block all emotion categories except for *neutral* were used for emotion induction. The motivation text to act out (and in the case of short sentences also to speak out) was shown on a computer screen along with the emotion labels the actors were instructed to portray. The actors went through the blocks in the described order, the order of emotion categories within blocks was randomized for each actor and block.

In the next three motion capture sessions each actor worked at one story a time. Each actor chose three stories out of the available 10, according to their own preference. Before the motion capture sessions each story was first split into utterances and annotated by the actors for eleven emotion categories. During the motion capture session the narration was shown utterance by utterance in its natural order and the emotion labels assigned by the actors were shown above each utterance. In both short scenarios and in full narrations, the actors were seated on a backless stool and progressed through short scenarios or full narrations by pressing a foot pedal, which allowed them to maintain their own speed and keep the upper body free for the expression of emotions. The timing of pedal presses was recorded for synchronization of acting script presentation and the motion capture data. The narrations were on average 300 utterances long (*M* = 298.5, *SD* = 36.09), each utterance containing a few word tokens. Each story annotation typically encompassed the full range of available emotion categories, yet the frequency between categories varied greatly, *neutral* naturally being the most frequent emotion and *shame* the least frequent.

The short scenarios are similar to classic motivation vignettes used for emotion induction in actors (see Bänziger and Scherer (2007) for a review of emotion induction methods). The actor portrays an emotion for a few seconds and then returns to a neutral pose. In contrast, during the narration task, the actors were immersed into the story and were displaying emotions in a way that was maximally close to natural day-to-day emotion expression. The actors were always free to express their emotions via face, voice, and body, their performance was also captured on video. Additionally, the plot and the word choice of the story contributed to the naturalness of emotion expression. Although fairy tales may seem a source of extreme and dramatic emotions, this impression mostly comes from the fact that the density (but not necessarily the intensity) of emotion instances in many fairy tales is indeed higher than in, e.g., novels (Mohammad, 2012) which still leaves them as a suitable textual material for our purposes because of their conciseness and clear identification of bad and good characters.

### 2.2. Hardware and software

**Figure 1:**
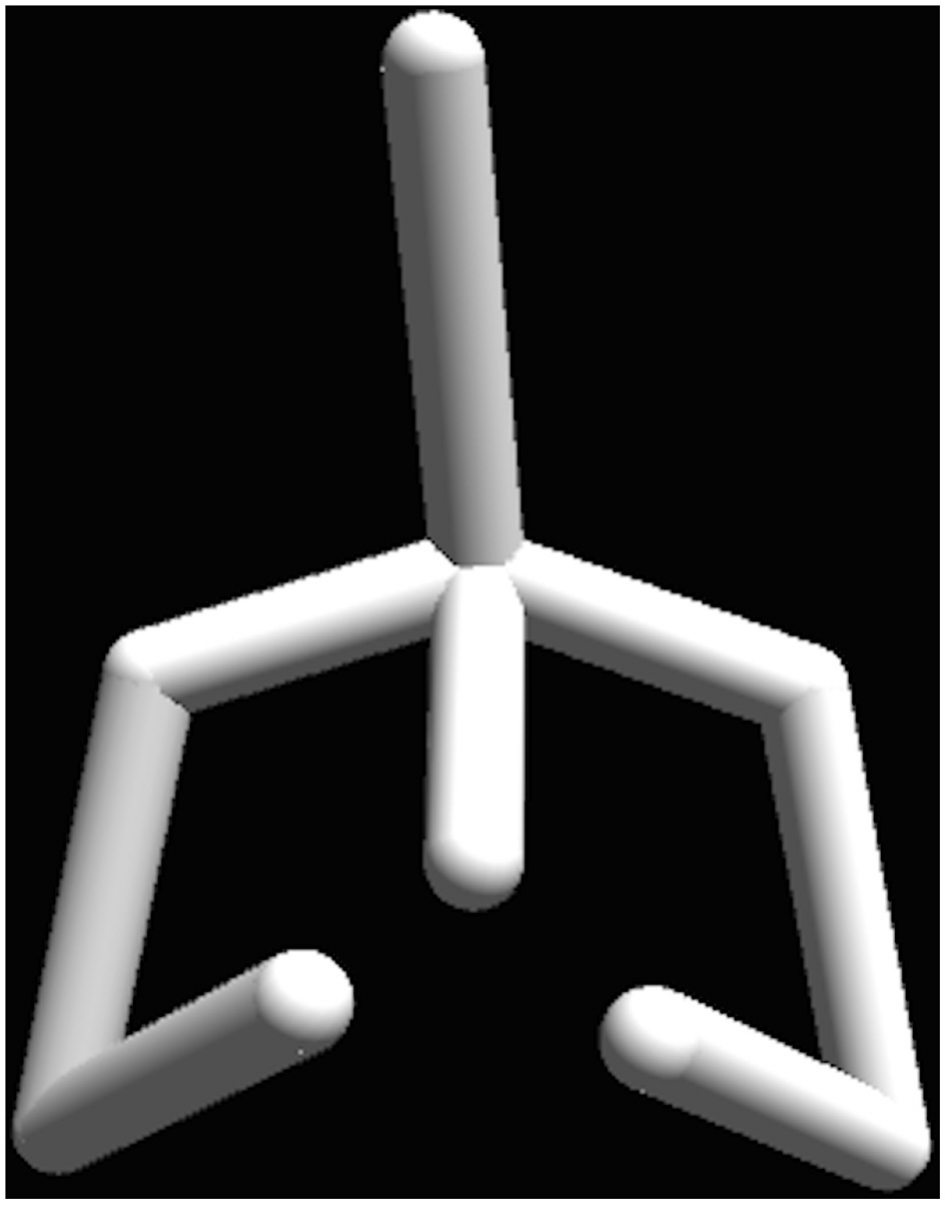
**The stick-figure stimuli display**. The pelvis and legs were excluded from the stimulus display, which includes only the upper body. The biological motion was displayed at 60 Hz (supported screen refresh rate) and came from real actors. The configuration of joins came from an underlying general skeleton model, keeping the body size, the proportions and other form cues the same for all stimuli.

The two experiments were programmed in the Unity 3D engine and ran on a MacBook Pro laptop. Two viewing conditions were used: a large screen with 102-inch in diagonal and a 17-inch laptop display. The participants were seated 1.5 m away from the large screen and 40 cm away from the laptop screen. In both experiments, the motion sequences were mapped onto a stick figure, which represented a human figure from the chest up, including the arms (Figure 1). The pelvis and legs were excluded from the stimulus display, since our main research question dealt with the upper body. The resulting stick figure displayed biological motion of the real actors, but its configuration came from the underlying general skeleton model and not from the actor. Thus the body size, the proportions and other form cues were kept the same for all stimuli. When using the large screen display the size of the stick figure was matched to the one of an average person and was adjusted to correspond to the height of the average person seated. The maximal horizontal visual angle for the stimuli on the large screen was 59°, corresponding to the maximum arm span of the stick figure (1.7 m) viewed from 1.5 m distance. In the laptop screen viewing condition the maximal horizontal visual angle was 19.3°.

### 2.3. General procedure and participants

In total, 87 volunteer participants were recruited: Table 2 gives the distribution of the participant numbers, gender and age across the experiments as well as viewing conditions. Informed written consent was obtained before every experiment session. Participants and the obtained data were treated strictly according to the Declaration of Helsinki. The experiments were approved by the local ethics committee of the University of Tübingen. All participants received monetary compensation for their participation, all had normal or corrected to normal visual acuity. None of the participants were aware of the purpose of the experiment. Due to the location of the experiment, many of the participants (58 out of 87) were German native speakers. All participants' command of English was sufficient to understand the instructions and the meaning of all the emotion categories used in the experiment.

**Table 2:** **Participants of the experiment**.

	**Participants**	**Age**	**Viewing condition**
Exp. 1	32 (16 f.)	*M* = 27.91, *SD* = 7.66	Laptop screen and large display
Exp. 2	55 (28 f.)	*M* = 29.96, *SD* = 9.96	Large display

*The columns show, from left to right: experiment number, number of participants and the gender distribution, participant age, and viewing conditions*.

General overview information for each experiment is presented in Table 3. Experiment 1 used only the 80 motion capture sequences from non-verbal solitary emotional scenarios (Table 1, column 2). The task for each of the 32 participants was to choose between 10 emotion categories, that is all categories mentioned in the beginning of Section 2 except for *neutral*, since no short scenarios included this category. Within each trial, the participant could always change their response before proceeding to the next trial. The motion sequence playback was set on the infinite playback loop, thus allowing the participants to watch the animation as many times as needed to perform the recognition task. Each of the animations was shown two times during the experiment. The trials were organized into two sessions and no animation occurred in one single session twice. The order of the animations was randomized for each session. To avoid fatigues, the participants took short 5-min breaks between the sessions.

**Table 3:** **Experiment setups**.

	**Stimuli**	**Playback**	**Trials**	**Duration**	**Categories**	**Acting tasks used**
Exp. 1	80	∞	160	≈ 1.5 h	10 emotions (all except *neutral*)	Nonverbal, solitary
Exp. 2	1700	3	340	≈ 3 h	11 emotions	All, full dataset

*The columns show, from left to right: experiment number, number of stimuli used in the experiment, animation playback (infinite or limited to 3 repetitions), number of trials in one experiment session, average session duration in hours, categories used, motions sequences source*.

The Experiment 2 used the full dataset of 1700 motion sequences, where 81% of the motion sequences come from narrations. Because no single participant could possibly categorize 1700 stimuli in one experiment session, the full dataset of motion sequences was organized into five equal blocks, making sure that the proportions of emotion categories were equal in each block. For each five participants the blocks were generated anew. Each participant categorized a unique block of 340 randomized motion sequences. Each of the motion sequences was seen by eleven participants throughout the experiment. Thus, our participant pool for this experiment amounted to 55 individuals.

In each of the 340 trials, motion sequence playback was set on three iterations, then the participant completed a two-fold response task and proceeded to the next trial. In the two-fold response task the participants were first asked to decide whether the animation carried any emotional information or whether it was completely *neutral*. If it was the former, the participant was to choose a specific emotion from the 10 remaining categories. The response could always be changed until the participant was satisfied and proceeded to the next trial. In order to avoid fatigue, short breaks of minimum 30 seconds were implemented after every 50 trials.

## 3. Results

### 3.1. Recognition accuracy

**Figure 2:**
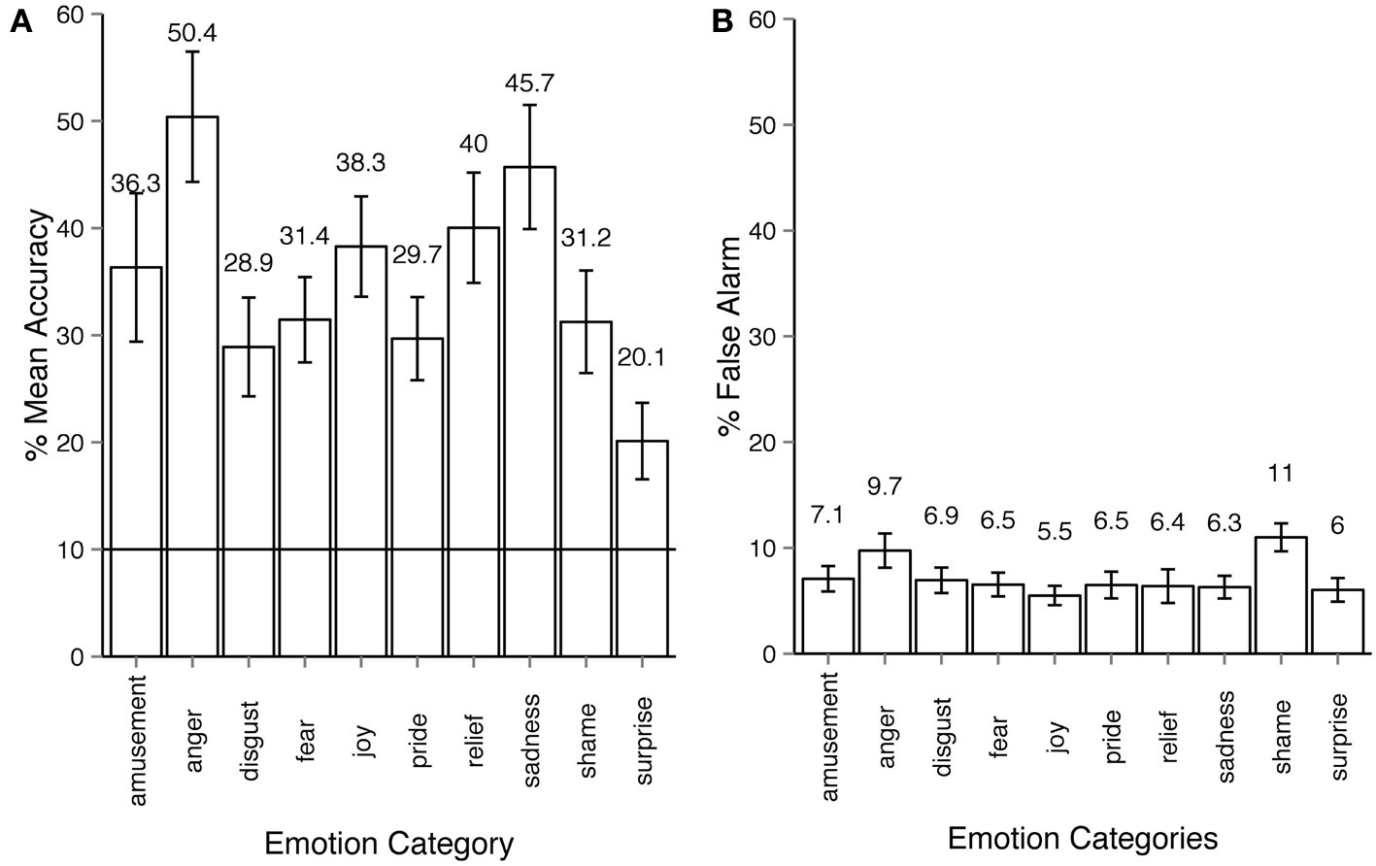
**Emotion recognition in experiment 1. (A)** Accuracy across emotion categories. The horizontal line shows the chance level threshold of 10%. **(B)** False alarm rate across emotion categories. All error bars show 95% confidence intervals. The participant observed a stick-figure representation of human upper-body motion. The task was to recognize the emotion category expressed by the actor and respond by choosing one of the buttons with the corresponding emotion category. The motion sequence was set on infinite loop playback, the participant could always alter their choice before proceeding to the next trial.

In Experiment 1, where only motion sequences from short non-verbal solitary scenarios were used, the average emotion recognition was 35.2% (Figure 2A). All emotion categories were correctly identified by participants on above chance level (10%), all Holm-corrected *p*-values are below 0.001 for one-tailed *t*-tests (exact values for recognition accuracy, *t*- and *p*-values for all experiments as well as an additional pilot study can be found in Supplementary materials, one-tailed *t*-tests are used since we are interested in recognition accuracy that is significantly higher than chance level). The between-participant factor of viewing condition had a non-significant effect on the recognition accuracy: 38% accuracy for large display condition vs. 31% accuracy in the desktop condition, ANOVA for viewing conditions: *F*_(1, 30)_ = 2.78, *p* = 0.10, η^2^_*p*_ = 0.021. The within-participants factor of emotion categories had a large effect on the recognition accuracy: *F*_(9, 270)_ = 15.41, *p* < 0.001, η^2^_*p*_ = 0.28. False alarm rates are at approximately 6% across all emotion categories with the exception of *shame* and *anger* (Figure 2B). This experiment established the upper threshold for emotion recognition in our dataset. It was unlikely that participants would be able to recognize the more subtle emotional body expressions taken from the narrations better than those from non-verbal scenarios.

**Figure 3:**
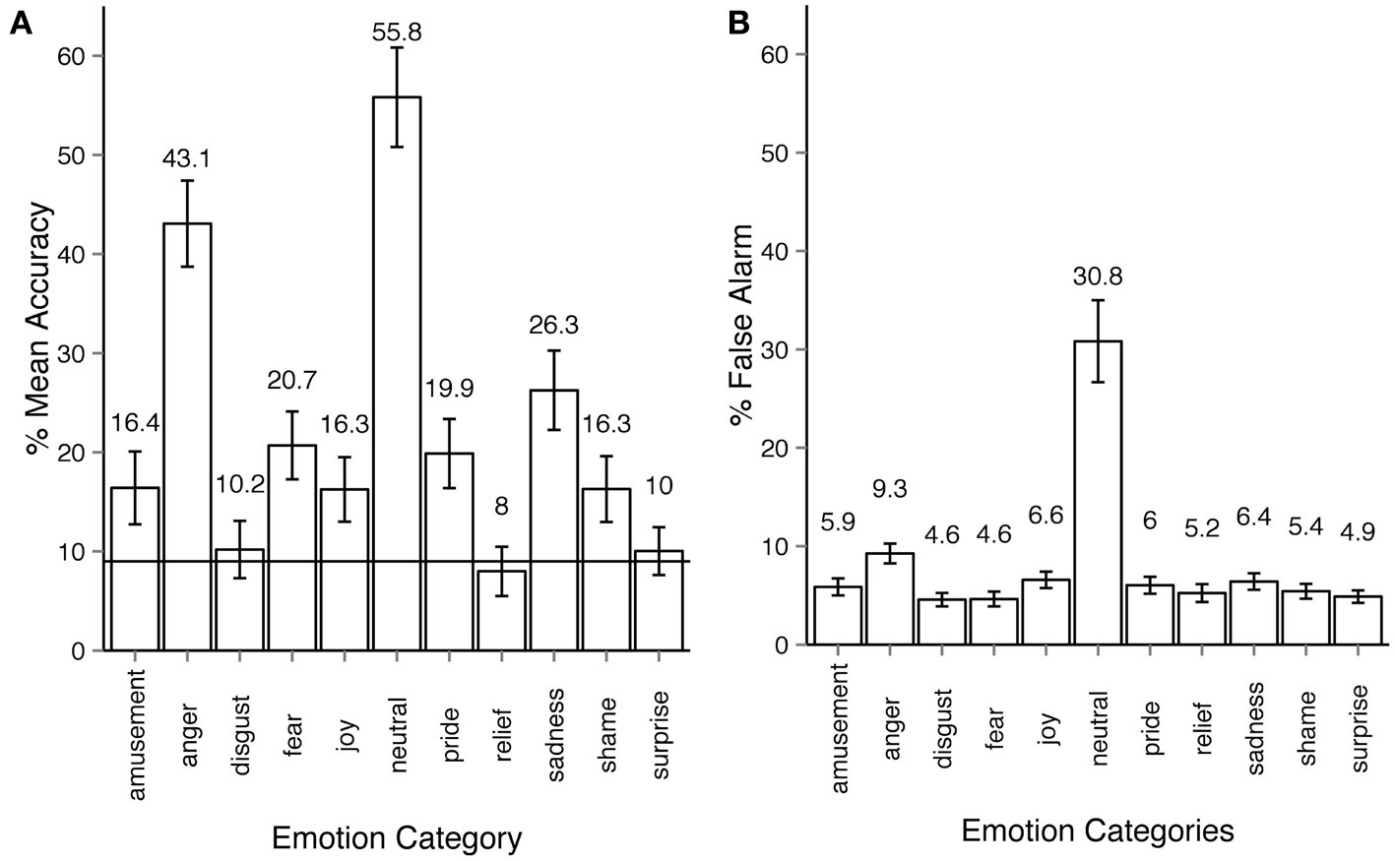
**Emotion recognition in experiment 2. (A)** Accuracy across emotion categories. The horizontal line shows the chance level threshold of 9%. **(B)** False alarm rate across emotion categories. All error bars show 95% confidence intervals. The participant observed a stick-figure representation of human upper-body motion. The task was to recognize the emotion category expressed by the actor and respond by first choosing either *neutral* or *emotional* category, then, if the *emotional* option was chosen, select the oppropriate emotion category of out ten non-neutral categories available. Each motion sequence was shown three times after which the participant had unlimited time to respond. The participant could always alter their choice before proceeding to the next trial.

The overall recognition rate for Experiment 2 was 18% (see Figure 3A). The majority of the emotion categories were correctly identified by participants on above chance level (9%), most Holm-corrected *p*-values are below 0.001 for one-tailed *t*-tests. However, three emotion categories, *disgust*, *relief*, and *surprise* were recognized at below chance level. Recognition accuracy was affected by the within-participant factor of expressed emotion category, ANOVA *F*_(10, 540)_ = 122, *p* < 0.001, η^2^_*p*_ = 0.68. False alarm rates across most emotions are similar to those in Experiment 1 (ca. 6%), with two important exceptions: *anger* (9.3%, almost the same rate as in Experiment 1 - 9.7%) and most importantly *neutral* with exceptionally high false alarm rate of 30.8% (Figure 3B).

**Figure 4:**
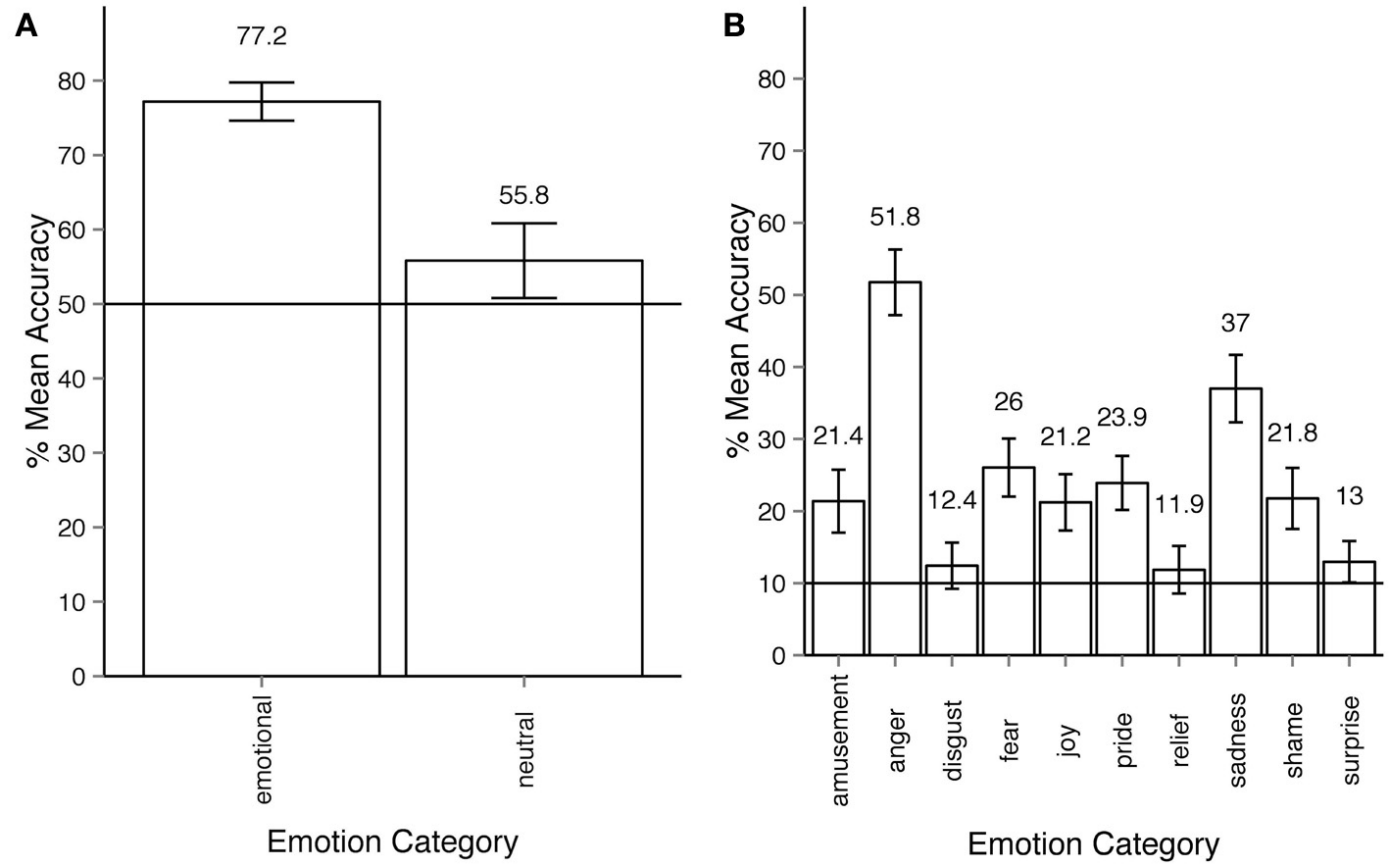
**(A)** Accuracy across emotion categories for experiment 2, response stage 1—*neutral* vs. *emotional*. The horizontal line shows the chance level threshold of 50%. **(B)** Accuracy across emotion categories for experiment 2, response stage 2—discrimination between all emotion categories except for *neutral*. The horizontal line shows the chance level threshold of 10%. All error bars show 95% confidence intervals.

In Experiment 2 observers' responses can also be analyzed according to the two-stage response structure they gave. In every trial the participant was first given the choice between *neutral* and *emotional*. If the participant considered the motion sequence to express an emotion other than *neutral*, the observer was to choose among the 10 remaining emotion categories. At any point of time within one trial the participant could change their response. In order to reflect the two-stage response structure, we ran two separate ANOVA's, one for the *neutral-emotional* level, the other for the 10 non-neutral categories. Figure 4A, shows that participants recognized whether a motion sequence was emotional or neutral at above chance level. The analysis of this first stage was performed on just two categories—*emotional* vs. *neutral*. The results show that the within-participant factor of emotionality had a significant effect on response accuracy: ANOVA *F*_(10, 540)_ = 9.86, *p* < 0.001, η^2^_*p*_ = 0.08. For the second stage of the response, the analysis of accuracy for 10 emotion categories (all except for *neutral*) as a within-participant factor shows that emotion categories had a significant effect on response accuracy (Figure 4B): ANOVA *F*_(9, 486)_ = 48.27, *p* < 0.001, η^2^_*p*_ = 0.44. Note that according to the two-stage analysis, all emotion categories were recognized at above chance level (50% for the first step, 10% for the second step of analysis), and all Holm-corrected *p*-values are below 0.05.

### 3.2. Inter-rater agreement and response consistency

Inter-rater agreement (IRA) gives a score of how much consensus there is in the ratings given by observers. This measure can be calculated in various ways, depending on the number of raters. When more than two raters are available, Fleiss' *kappa* (Fleiss, 1971) is often used to measure IRA. However, since in our experiment the observers operated with non-parametric data ratings (emotion categories) we used Kendall's coefficient of concordance (Kendall and Smith, 1939) to obtain an estimate for IRA. Kendall's W ranges from 0.0 (no agreement) to 1.0 (complete agreement). In Experiment 1 *W* is equal to 0.26 (averaged across the two experiment sessions) and in Experiment 2 *W* is equal to 0.24 (see Table 4). IRA measures general consensus among the annotators and is not useful for calculating agreement for each motion sequence.

**Table 4:** **Inter-rater agreement in two experiments according to Kendall's coefficient of concordance *W***.

	**Raters**	**Categories**	**Items**	**IRA *(W)***
Exp. 1	32	10	80	0.26
Exp. 2	11	11	1700	0.24

*The columns show, from left to right: number of raters (participants) per motion sequence, number of categories available for categorization, number of items to categorize and the inter-rater agreement*.

**Figure 5:**
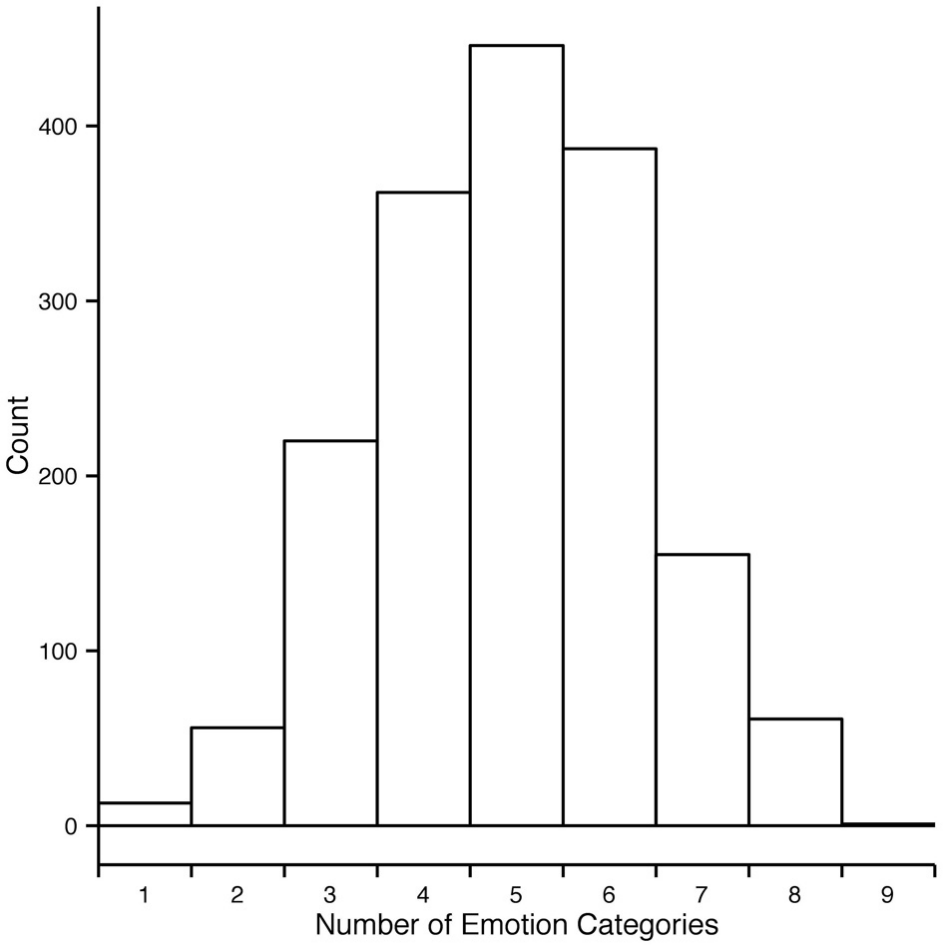
**Distribution of number of distinct categories given to each motion sequence in experiment 2**. Each motion sequence received in total eleven responses. For most motion sequences the responses fall into five or fewer categories.

**Figure 6:**
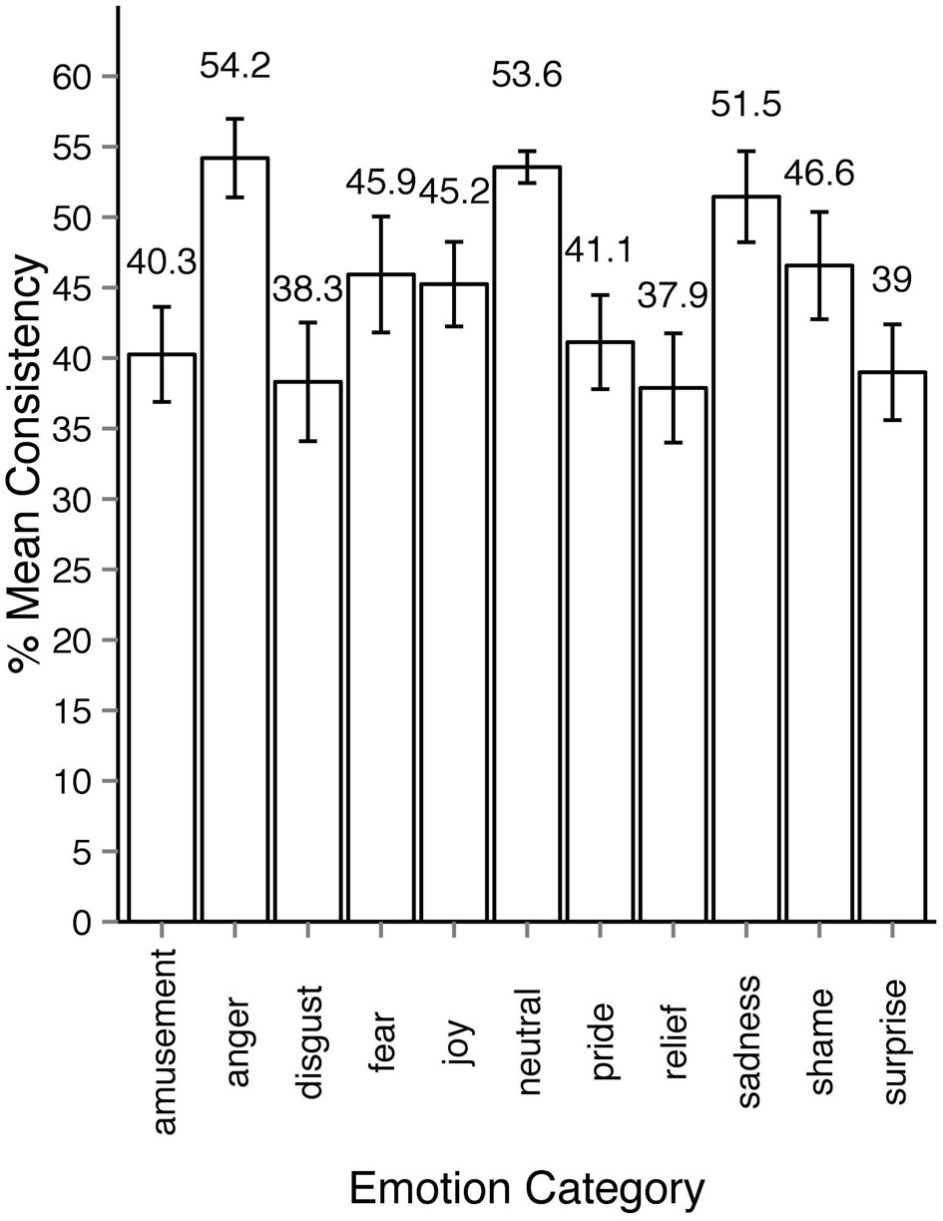
**Average consistency levels across emotion categories in experiment 2**. The sequences are assigned emotion labels according to the modal category in observers response distribution. All error bars show 95% confidence intervals.

We thus developed an alternative measure for estimating agreement among observers and will henceforth refer to it as consistency (*c*). Consistency is the percentage of observers' responses falling into a particular emotion category that forms the modal value in the response distribution. For example, if for a specific motion sequence all responses fall into one category, *c* = 100%. If three out of 10 responses fall into one category and no other category received three responses or more, *c* = 30%. The minimally possible *c* is always 100 divided by the number of observations for the given stimulus and multiplied by 2 because there have to be at least least two observers assigning the same category to the stimuli to form a modal value. In cases when the response distribution is bimodal or multimodal, *c* cannot be defined. In the rest of the section we will focus on the consistency results of Experiment 2 since it encompassed all 1700 motion sequences. As Figure 5 shows, most responses to animations were distributed among five or fewer emotion categories. Regardless of the intended emotion of the actor, for 1452 motion sequences (85% of 1700) observers preferred one emotion category for a given motion sequence over other categories. Moreover, in 40% of the cases (670 out of 1700 motion sequences) the modal value received more than half of the responses (at least six out of eleven responses). Figure 6 shows the levels of consistency across all emotion categories, most of which are above 40%.

### 3.3. Response bias

Bias in the categorization of motion sequences into two emotions (*anger* and *neutral*) was observed in Experiment 2. In Experiment 1 the *neutral* category was not used, but the recognition accuracy of *anger*, though highest among other categories, is not significantly different from *sadness* (*p* = 1.0) and relief (*p* = 0.08) according to Holm-corrected pairwise comparison. In Experiment 2 both *neutral* and *anger* are significantly different from all other emotions—all *p*-values are below 0.001 with the exception of the comparison between *anger* and *neutral* themselves where *p* = 0.004, thus still significantly different between each other.

In Experiment 2, the *neutral* category was chosen more frequently than other categories. The frequency of *neutral* responses was 34% on average, several times more than the actual number of *neutral* animations in the dataset (7%). Indeed, the results of Experiment 2 showed that the observers often made mistakes in recognizing an emotion when deciding whether the motion sequence conveyed any emotion at all. Confusions between two *non-neutral* categories were less frequent and rather systematic (see Section 3.4 for more detail). By comparing Figures 3, 4 one can see that once the *emotional* vs. *neutral* stage is separated from the second stage of response, all emotion categories were recognized above chance. False alarm rate contributed important information concerning the observers' response patterns. As the right side bar plots of Figures 2, 3 show, false alarm rates for most categories lie under 10%. In Experiment 2 however, the *neutral* category clearly received more false alarms than other emotion categories (30.8%). This means that in many cases when an emotion category other than *neutral* was intended by the actor, it was nevertheless perceived as *neutral* by the observers. The reasons behind the bias toward *neutral* and *anger* are discussed in Section 4.

### 3.4. Motion properties

**Figure 7:**
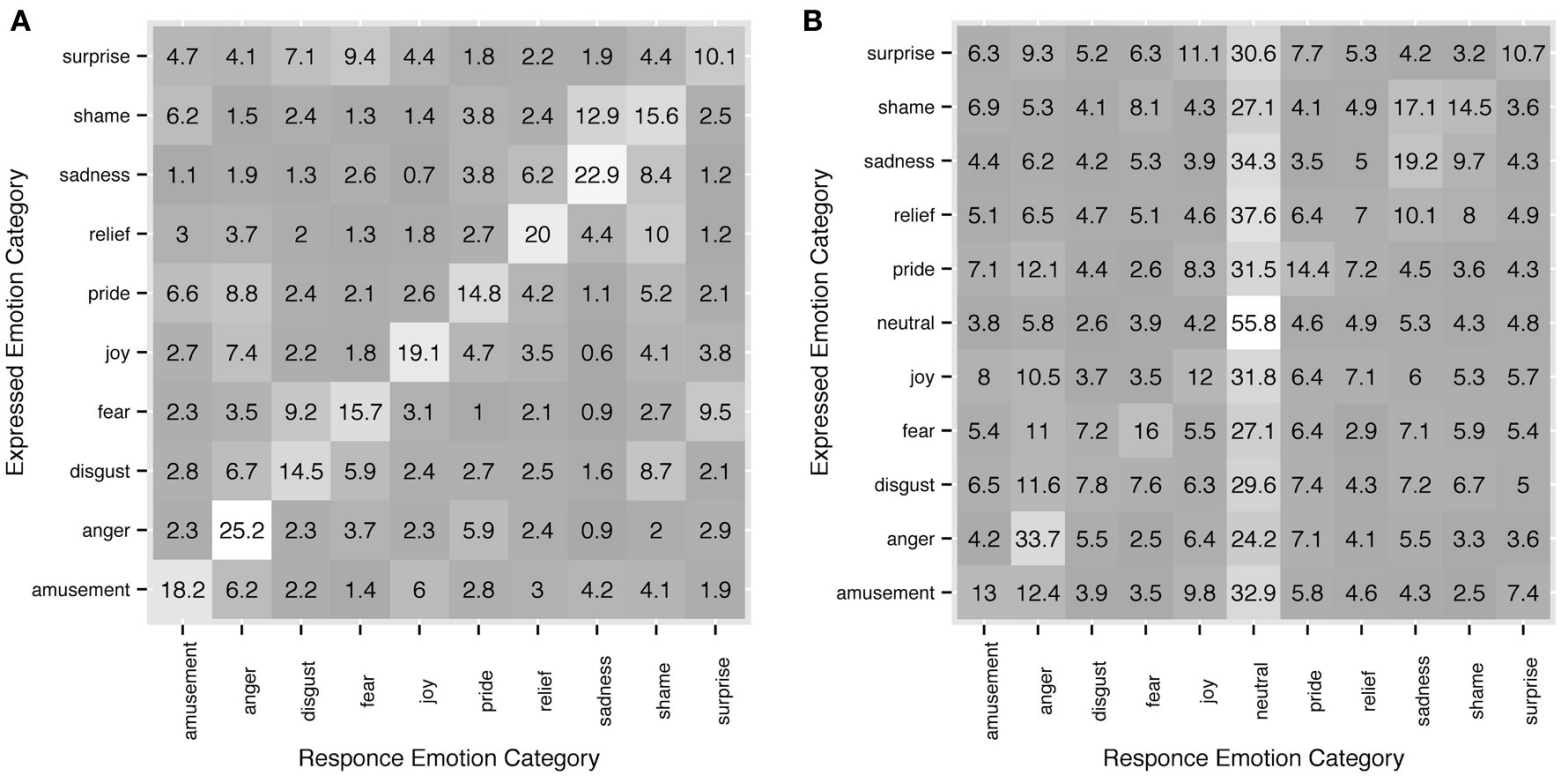
**Detailed correspondence between intended emotion categories and observers' responses in experiment 1 (A) and experiment 2 (B)**. On *y* axis the intended categories are shown, on *x* axis - the response categories. The diagonal shows response accuracy. Each row sums up to 100. All values are in %.

Recognition rates, inter-rater agreement and response consistency all clearly show that observers' choices of response categories are not random. When an observer failed to recognize an intended emotion from a motion sequence, there was a tendency for other categories to occur in the responses depending on the actor's intentions. For instance, in Experiment 1, *sadness* was accurately recognized in 45.7% of the trials, and the 54.3% errors are distributed unevenly among the rest of emotion categories with 16% falling into the category of *shame*. Shame on the other hand was accurately recognized in 31.2% of the trials and in 25.8% it was categorized as *sadness*. Figure 7 shows more examples and full detail on distribution of response categories across intended emotions. Since upper-body motion was all the information available to the observers, commonalities in motion patterns between different categories could possibly explain confusions between intended emotion categories and observers' responses.

Motion analysis should provide a way to compare motion sequences that have been stably labeled for certain emotion categories. Similarities and differences between motion sequences can be looked for at different levels. At a semantic level, meaningful patterns and gestures like shaking fists in anger, crossing arms and tilting head when expressing pride and clapping hands for joy can be considered. On the other hand, each motion sequence can be described as a set of more descriptive statistics, e.g., speed, peaks in motion trajectory, span of motion, duration and so on. Compare clapping your hands in a fast energetic manner to express joy and clapping your hands slowly to express contempt, cold anger or disgust. The motion trajectories are the same yet motion properties like speed and span differ depending on what emotion is expressed. Extreme joy and hot anger are most likely recognized by their fast, broad motion, while the motion profile of sadness is notable for its low speed.

**Figure 8:**
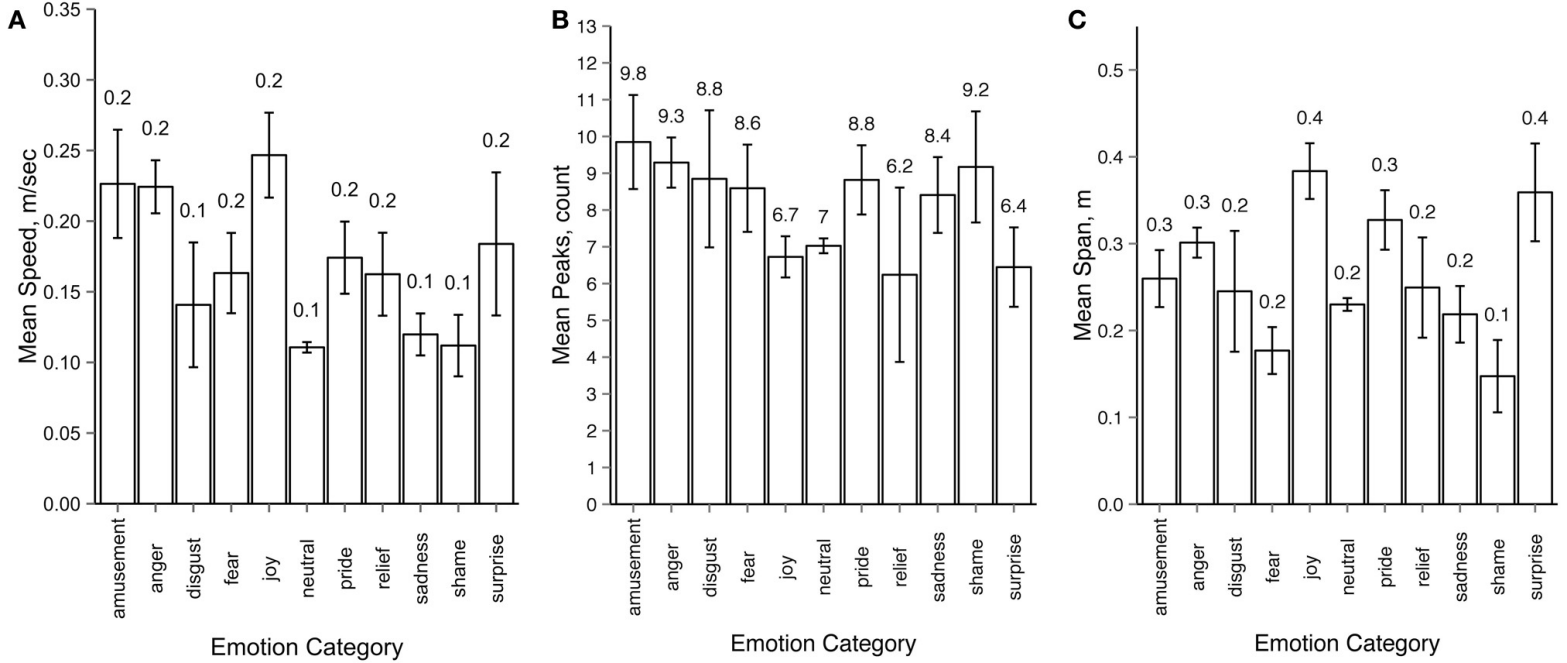
**Average values for (A) motion speed (m/sec), (B) peaks (raw count) and (C) span (m) across emotion categories**. Modal values of the response distributions were used for grouping motion sequences into emotion categories. All error bars show 95% confidence intervals.

To test whether motion properties can predict response categories, we ran a multiple regression analysis using response categories as the dependent variable and several extracted motion properties as independent variables. For motion properties extraction we used only the left and the right wrist joints of the underlying body structure, because in our setup they were the most mobile joints for all emotions. For each motion sequence we calculated the mean motion speed, the average number of peaks in the trajectories for the *x, y* and *z* axes and the mean span of the motion, defined as average distance between the wrist joints during the motion sequence. For the purpose of this particular analysis the intended emotions from the actors could not be used, since we aimed to gain insight behind the observers' decision during the recognition tasks. Thus for each motion sequence we first established the distribution of response labels across emotion categories, similar to the response consistency analysis described in Section 3.2. Only motion sequences with a unique modal value were included in the analysis (78 out of 80 for Experiment 1 and 1452 out of 1700 in Experiment 2), where the emotion category representing the response modal value was used as the label for each motion sequence. Figure 8 gives an overview of the resulting values for mean motion speed (A), peak count (B) and mean span (C).

In order to perform the analysis we have calculated a distance matrix for response patterns for intended emotions and distance matrices for each of the motion properties. We tested if motion properties (speed, peaks, and span) significantly predicted response based distance between emotions for both experiments. The results of the regression analysis for Experiment 1 indicated that the three predictors explained 48.3% (adjusted *R*^2^) of the variance [*R*^2^ = 0.48, *F*_(3, 96)_ = 29.99, *p* < 0.001]. Mean motion speed significantly predicted emotion distance (β = 0.35, *p* < 0.001), as did number of peaks (β = 0.32, *p* < 0.001), and mean motion span (β = 0.36, *p* < 0.001). The results of the regression analysis for Experiment 2 indicated the three predictors explained 34.9% (adjusted *R*^2^) of the variance [*R*^2^ = 0.36, *F*_(3, 117)_ = 22.53, *p* < 0.001]. The distance in mean motion speed across emotions significantly predicted emotion distance (β = 0.20, *p* = 0.014), as did number of peaks (β = 0.31, *p* < 0.001), and mean motion span (β = 0.33, *p* < 0.001). The assumptions of independence and multicollinearity were met for all predictors in both experiments.

## 4. Discussion

The experiments presented in this paper demonstrate that people recognize naturally expressed emotions with only upper body motion available. Since the motion sequences come from a naturalistic narration setting and the visual stimuli only provide information about the upper body motion, the recognition rate is impressive. In Experiment 1 the recognition is high across all 10 emotion categories. This can be contributed to the fact that the motion sequences used in this experiment came from non-verbal solitary short scenarios, so more information was likely conveyed in the motion of the body since the auditory channel was not used. In Experiment 2 seven out of ten emotions are recognized at above chance level despite the fact that most of the motion sequences used in this experiment came from narration tasks where all expressive channels were used by the actors. Not only is the recognition above chance but motion sequences were categorized with high consistency between participants. For 85% of the motion sequences response distribution has a unique modal value and for 40% of all motion sequences the majority (over 50%) of the observers' responses fall into one category.

Notably, recognition accuracy and consistency levels differ among emotion categories. Specifically, observers have a bias for categorizing emotions as *anger* in both experiments, most prominently in Experiment 2. Recognition and false alarm rates, as well as high consistency rates for *anger* show that people are prone to categorize motion sequences with *anger* more often than other categories, with the exception of *neutral* in Experiment 2. A possible explanation for the bias toward *anger* categorization could be the evolutionary importance of detecting anger as a potential threat regardless of which channel (language, prosody, face, or body) it is perceived through. Several previous works support the importance of anger expressed via body motion: Pichon and colleagues have shown that a response to anger expression results in even more activation of defense mechanisms (“fight or flight”) than a response to fear expressions when the perceiver is the target of the anger (Pichon et al., 2009). A similar tendency is discussed in a recent study by Visch et al. (2014), where recognition of anger expressed in the body motion was the most robust under various stimuli degradation conditions (including a condition where only parts of the upper body were portrayed). Others have found that a bias toward anger is even more pronounced in violent offenders (Kret and Gelder, 2013).

Moreover, participants have a bias toward the *neutral* category in Experiment 2, as the *neutral* category also has high accuracy rate, false alarm rate and consistency levels. A likely reason for this bias is that many motion sequences did not possess properties that could communicate any particular emotion to the observer, as is supported by the high false alarm rate.

In order to gain insight into errors in emotion recognition of the intentions of the actor and factors behind them, we analyzed the relationship between the intended emotions, the responses, and motion properties of the motion sequences. We found that distances in mean motion speed, number of peaks and mean span between motion sequences stably marked for certain emotions can to some extent predict distances between response categories. These findings do not belittle the significance of meaningful motion trajectories and gestures, e.g., fist shakes, hand claps and head nods. However, many motion properties, e.g., motion speed, are easier to extract from any biological motion than specific gestures. These findings are encouraging for future work in automatic emotion recognition and are in agreement with related research. Huis in 't Veld et al. (2014) found that both active expression and passive viewing of emotions via body motion activate similar muscle groups in the upper body. Interestingly, a study by Magnée et al. (2007) showed emotion specific facial muscle activity that was independent of stimuli type (facial expressions, bodily expressions or face-voice combinations). It would be intriguing to investigate whether spontaneous reaction to emotional stimuli in the body is as modality independent as the one observed in the face.

Our results also provide some evidence that using a rich set of emotions that goes beyond the basic Ekman emotions for body motion recognition is valuable. One of the major arguments for basic emotions (Ekman, 1992) is that they are saliently recognized in most populations regardless of age, gender or culture and are independent of expression medium (face, body, voice). However, the recognition rates in our experiments seem to suggest that for emotional body expressions in a natural setting the basic Ekman emotions are not sufficient. In Experiment 2, two out of three categories recognized below chance are basic: *disgust* and *surprise*, while non-basic emotions of *amusement*, *pride* and *shame* are recognized above chance. However, all emotions, basic and non-basic, are recognized well above chance in Experiment 1, where motion sequences were obtained from purely non-verbal short scenarios. This allows us to conclude that the *distinctive universal signals* proposed by Ekman as one of the characteristics for basic emotions are not always present in our upper body motion patterns captured during natural expression.

## 5. Conclusion

Body motion is an important source of information in emotion expression. This research adds a novel approach by focusing on the perception of emotional body language occurring naturally in narrative scenarios. We used a rich set of eleven emotion categories in two perceptual experiments that investigated emotion recognition of upper body movements on stick figure stimuli. Almost all emotion categories achieved recognition accuracy that surpassed the chance level (ranging from 8 to 58%). Response consistency between the participants is strong, as for most motion sequences the distribution of response categories has a unique modal value, meaning that most observers chose one category as their response. Further, in 40% of the motion sequences more than half of the participants agreed on this modal value. In our experiments there is a strong bias for the *anger* category among observers' responses. This can be explained by the ecological importance of early anger detection in human environment as one of the survival strategies of fight or flight. There was additionally a strong bias toward *neutral*, which might be due to the low amount of movement in natural scenarios. In order to further consider what factors were driving the errors in recognition performance, we performed a multiple regression analysis using the descriptive statistics of motion, namely speed, peaks in motion trajectory and span. Our findings show that the investigated motion properties can serve as predictors for patterns in response categories. Overall, the results demonstrate that the information contained in upper body motion in natural scenarios is enough for people to recognize emotion.

## Author contributions

Ekaterina P. Volkova, Betty J. Mohler, and Joachim Tesch designed the study. Ekaterina P. Volkova acquired the data. Ekaterina P. Volkova, Betty J. Mohler and Trevor J. Dodds analyzed the data. Ekaterina P. Volkova, Betty J. Mohler, Trevor J. Dodds, Joachim Tesch, and Heinrich H. Bülthoff wrote the article. All authors reviewed the article and approved its publication.

## Funding

Part of Heinrich Bülthoff's research was supported by the Brain Korea 21 PLUS Program through the National Research Foundation of Korea funded by the Ministry of Education.

### Conflict of interest statement

The authors declare that the research was conducted in the absence of any commercial or financial relationships that could be construed as a potential conflict of interest.
