# Supplementary material for: Emotion categorization of body expressions in narrative scenarios
Source: Front Psychol. 2014 Jun 30;5:623. doi: 10.3389/fpsyg.2014.00623 (PMC4075474; doi:10.3389/fpsyg.2014.00623)
Supplement: Supplementary file 2 [file Presentation1.PDF]

Frontiers in Psychology

Research Article
9 May 2014

Supplementary Material: Emotion Categorisation of Body Expressions in Narrative Scenarios

Ekaterina P. Volkova [1,2,*], Betty J. Mohler [1], Trevor J. Dodds [1], Joachim Tesch [1] and Heinrich H. Bülthoff [1,3,*]

[1] *Human Perception, Cognition and Action, Max Planck Institute for Biological Cybernetics, Tübingen, Germany*
[2] *Graduate School of Neural & Behavioural Sciences, Tübingen, Germany*
[3] *Department of Brain and Cognitive Engineering College of Information and Communication, Korea University, Seoul, Korea*

Correspondence*:
Ekaterina P. Volkova and Heinrich H. Bülthoff
Human Perception, Cognition and Action
Max Planck Institute for Biological Cybernetics
Spemannstr. 38,
72076, Tübingen, Germany,
{ekaterina.volkova,heinrich.buelthoff}@tuebingen.mpg.de

1 PILOT STUDY

We conducted a pilot study on a subset of motion sequences. The pilot study employed motion sequences from the whole dataset (non-verbal expressions, short sentences and narrations), but for every emotion category only 20 motion sequences were randomly selected and used throughout the study, amounting to 220 stimuli in total for each participant. The number of animations for each emotion was balanced in order to discover any possible response bias towards emotion categories. In each trial the participant could choose between eleven emotion categories (*amusement, joy, pride, relief, surprise, anger, disgust, fear, sadness, shame, neutral*) or the *cannot identify* response option in case they found themselves unable to categorise the stimuli. Within each trial, the participant could always change their response before proceeding to the next trial. Each motion sequence was used once during the experiment, in each trial the motion sequence was played back three times. The order of stimuli was randomise for each participant.

Twelve participants took part in the pilot study (7 female, age: M=26.16, SD=5.63) The stimuli were presented on a 17-inch laptop display, the participants viewed the stimuli from 40 cm distance. The recognition rate in the pilot study was 15% which was nevertheless above chance level of 9% (based on 11 possible categories). Table 5 reports recognition accuracy, as well as t- and p-values for the recognition above chance level for each emotion category. According to Holm-corrected p-values, only four emotion categories, namely *anger*, *fear*, *neutral*, and *sadness* were recognised at above chance level. The response option of *cannot identify* was used rarely, on average only in 3.6% of the cases, no single category of intended emotion received this label more often than other categories. Similarly to Experiments 1, emotion categories had a significant effect on the overall recognition rate, ANOVA $F(10, 110) = 8.81, p < .001, \eta_p^2 = 0.43$. Table 9 shows pairwise t-test comparisons with Holm-corrected p-values as post-hoc analysis.

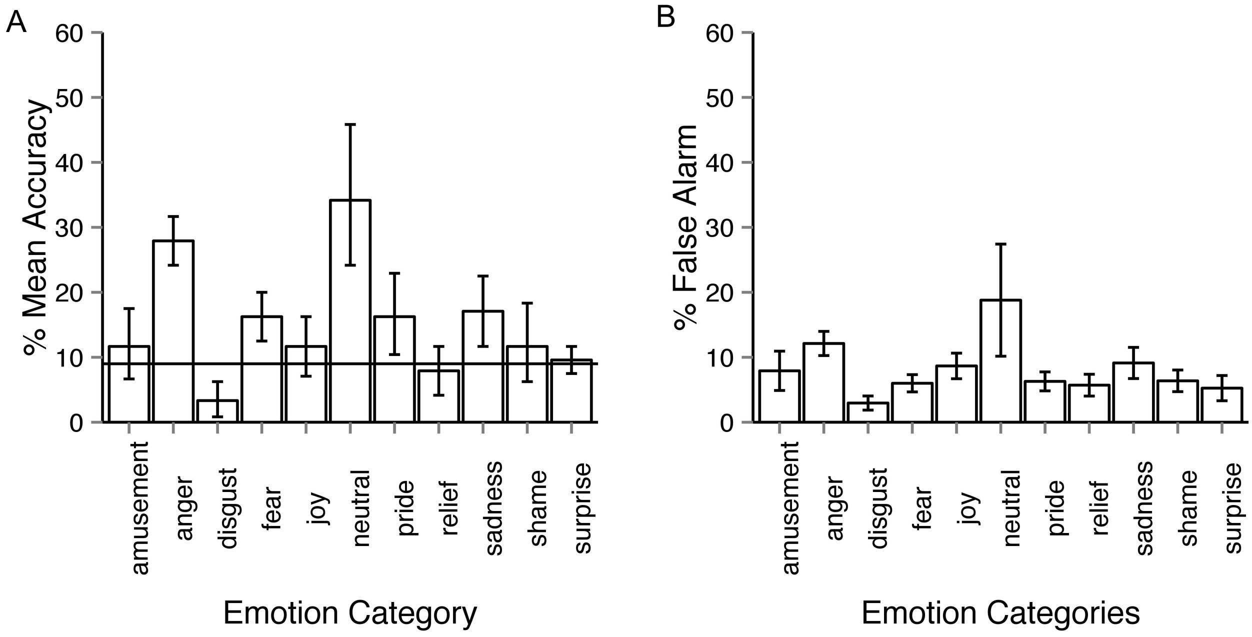

Supplementary Figure 1. Emotion recognition in experiment 2. A: Accuracy across emotion categories. The horizontal line shows the chance level threshold of 9%. B: False alarm rate across emotion categories. All error bars show 95% confidence intervals.

Two major observation can be made on the basis of the pilot study results. First, despite the fact that each category had an equal number of representative motion sequences, there is obvious bias towards *anger* and *neutral* categories, which is reflected in the recognition accuracy rates and the false alarm rates (see Figure 1). Second, since the *neutral* category has a high false alarm rate, it could be interpreted as a default category the observers use when they are uncertain in what emotion is expressed in the stimuli. While the distinction between *neutral* and *absence of any emotion* is vague, the most likely explanation for high false alarm rates for neutral is the subtlety of emotional expression in those motion sequences. This is supported by the fact that in the pilot study the cannot identify option was available and yet barely used. Based on these results in Experiments 1 and 2 in the manuscript we did not use a *cannot identify* option.

2 SUPPLEMENTARY TABLES AND FIGURES

Supplementary Table 1. Experiment 1, recognition accuracy for each emotion category with t and Holm-corrected p-values for performance above chance level (μ).

Emotion	Acc., %	$t, \mu = 0.1$	p_t
amusement	36	7.75	$< .001$
anger	50	13.54	$< .001$
disgust	28	8.37	$< .001$
fear	31	10.97	$< .001$
joy	38	12.33	$< .001$
pride	29	10.34	$< .001$
relief	40	11.90	$< .001$
sadness	45	12.57	$< .001$
shame	31	9.05	$< .001$
surprise	20	5.79	$< .001$

Supplementary Table 2. Experiment 2, recognition accuracy for each emotion category with t and Holm-corrected p-values for performance above chance level (μ)

Emotion	Acc., %	$t, \mu = 0.09$	p_t
amusement	16	3.99	.001
anger	43	15.49	$< .001$
disgust	10	0.81	.59
fear	20	6.74	$< .001$
joy	16	4.41	$< .001$
neutral	55	18.74	$< .001$
pride	19	6.14	$< .001$
relief	7	-0.79	.78
sadness	26	8.53	$< .001$
shame	16	4.33	$< .001$
surprise	10	0.84	.59

Supplementary Table 3. Experiment 2, recognition accuracy for *neutral* category and the remaining ten categories combined with t and Holm-corrected p-values for performance above chance level (μ)

Category	Acc., %	$t, \mu = 0.5$	p_t
non-neutral	77	20.89	$< .001$
neutral	55	2.32	.01

Supplementary Table 4. Experiment 2, recognition accuracy for all *emotional* categories except for *neutral* with t and Holm-corrected p-values for performance above chance level (μ)

Category	Acc., %	$t, \mu = 0.1$	p_t
amusement	21	5.60	$< .001$
anger	51	18.54	$< .001$
disgust	12	2.11	.03
fear	26	8.36	$< .001$
joy	21	6.17	$< .001$
pride	23	7.86	$< .001$
relief	11	1.70	.04
sadness	36	11.82	$< .001$
shame	21	5.94	$< .001$
surprise	12	2.72	.01

Supplementary Table 5. Pilot study, recognition accuracy for all emotion categories with t and Holm-corrected p-values for performance above chance level (μ)

Emotion	Acc., %	$t, \mu = 0.09$	p_t
amusement	11	0.89	0.97
anger	27	9.50	$< .001$
disgust	3	-3.98	1.00
fear	16	3.5	.02
joy	11	1.10	0.87
neutral	34	4.26	$< .006$
pride	16	2.18	.18
relief	7	-0.54	1.00
sadness	17	2.97	.05
shame	11	0.81	.97
surprise	9	0.50	.97

Supplementary Table 6. Experiment 1 post-hoc analysis, pairwise t-tests with Holm-corrected p-values

	amusement	anger	disgust	fear	joy	pride	relief	sadness	shame
anger	0.00235	-	-	-	-	-	-	-	-
disgust	0.62488	1.1e-07	-	-	-	-	-	-	-
fear	1.00000	4.8e-06	1.00000	-	-	-	-	-	-
joy	1.00000	0.01905	0.18660	0.77481	-	-	-	-	-
pride	0.82124	3.7e-07	1.00000	1.00000	0.29204	-	-	-	-
relief	1.00000	0.08997	0.04679	0.29204	1.00000	0.08997	-	-	-
sadness	0.18660	1.00000	9.1e-05	0.00193	0.62488	0.00024	1.00000	-	-
shame	1.00000	3.7e-06	1.00000	1.00000	0.72584	1.00000	0.27551	0.00159	-
surprise	0.00019	1.3e-14	0.27551	0.04012	1.4e-05	0.16494	1.2e-06	9.9e-11	0.04679

Supplementary Table 7. Experiment 2 post-hoc analysis, pairwise t-tests with Holm-corrected p-values

	amusement	anger	disgust	fear	joy	neutral	pride	relief	sadness	shame
anger	<2e-16	-	-	-	-	-	-	-	-	-
disgust	0.18017	<2e-16	-	-	-	-	-	-	-	-
fear	0.84967	<2e-16	0.00045	-	-	-	-	-	-	-
joy	1.00000	<2e-16	0.18738	0.84967	-	-	-	-	-	-
neutral	<2e-16	0.00437	<2e-16	<2e-16	<2e-16	-	-	-	-	-
pride	1.00000	<2e-16	0.00164	1.00000	1.00000	<2e-16	-	-	-	-
relief	0.01086	<2e-16	1.00000	4.4e-06	0.01292	<2e-16	2.6e-05	-	-	-
sadness	0.00118	1.6e-10	1.2e-09	0.28530	0.00098	<2e-16	0.16453	1.4e-12	-	-
shame	1.00000	<2e-16	0.18738	0.84967	1.00000	<2e-16	1.00000	0.01276	0.00102	-
surprise	0.16453	<2e-16	1.00000	0.00032	0.18017	<2e-16	0.00122	1.00000	7.1e-10	0.18017

Supplementary Table 8. Experiment 2 post-hoc analysis for all *emotional* categories except for *neutral*, pairwise t-tests with Holm-corrected p-values

	amusement	anger	disgust	fear	joy	pride	relief	sadness	shame
anger	<2e-16	-	-	-	-	-	-	-	-
disgust	0.00170	<2e-16	-	-	-	-	-	-	-
fear	1.00000	<2e-16	3.5e-05	-	-	-	-	-	-
joy	1.00000	<2e-16	0.05650	0.73816	-	-	-	-	-
pride	1.00000	<2e-16	0.00041	1.00000	1.00000	-	-	-	-
relief	0.00077	<2e-16	1.00000	1.3e-05	0.02897	0.00016	-	-	-
sadness	1.3e-05	7.0e-11	<2e-16	0.00077	5.5e-08	7.4e-05	<2e-16	-	-
shame	1.00000	<2e-16	0.00124	1.00000	1.00000	1.00000	0.00056	1.9e-05	-
surprise	0.33939	<2e-16	1.00000	0.02685	1.00000	0.13302	0.74656	1.6e-11	0.28664

Supplementary Table 9. Pilot study post-hoc analysis, pairwise t-tests with Holm-corrected p-values

	amusement	anger	disgust	fear	joy	neutral	pride	relief	sadness	shame
anger	0.00610	-	-	-	-	-	-	-	-	-
disgust	1.00000	1.5e-06	-	-	-	-	-	-	-	-
fear	1.00000	0.20677	0.08714	-	-	-	-	-	-	-
joy	1.00000	0.00610	1.00000	1.00000	-	-	-	-	-	-
neutral	1.4e-05	1.00000	8.1e-10	0.00141	1.4e-05	-	-	-	-	-
pride	1.00000	0.20677	0.08714	1.00000	1.00000	0.00141	-	-	-	-
relief	1.00000	0.00019	1.00000	1.00000	1.00000	2.1e-07	1.00000	-	-	-
sadness	1.00000	0.34718	0.04677	1.00000	1.00000	0.00293	1.00000	0.96890	-	-
shame	1.00000	0.00610	1.00000	1.00000	1.00000	1.4e-05	1.00000	1.00000	1.00000	-
surprise	1.00000	0.00096	1.00000	1.00000	1.00000	1.5e-06	1.00000	1.00000	1.00000	1.00000
